# Digitally deconstructing leaves in 3D using X‐ray microcomputed tomography and machine learning

**DOI:** 10.1002/aps3.11380

**Published:** 2020-07-31

**Authors:** Guillaume Théroux‐Rancourt, Matthew R. Jenkins, Craig R. Brodersen, Andrew McElrone, Elisabeth J. Forrestel, J. Mason Earles

**Affiliations:** ^1^ Institute of Botany University of Natural Resources and Life Sciences Vienna Austria; ^2^ Department of Viticulture and Enology University of California Davis California USA; ^3^ School of Forestry and Environmental Sciences Yale University New Haven Connecticut USA

**Keywords:** image segmentation, microCT, plant leaf internal anatomy, plant phenotyping, random forest classification

## Abstract

**Premise:**

X‐ray microcomputed tomography (microCT) can be used to measure 3D leaf internal anatomy, providing a holistic view of tissue organization. Previously, the substantial time needed for segmenting multiple tissues limited this technique to small data sets, restricting its utility for phenotyping experiments and limiting our confidence in the inferences of these studies due to low replication numbers.

**Methods and Results:**

We present a Python codebase for random forest machine learning segmentation and 3D leaf anatomical trait quantification that dramatically reduces the time required to process single‐leaf microCT scans into detailed segmentations. By training the model on each scan using six hand‐segmented image slices out of >1500 in the full leaf scan, it achieves >90% accuracy in background and tissue segmentation.

**Conclusions:**

Overall, this 3D segmentation and quantification pipeline can reduce one of the major barriers to using microCT imaging in high‐throughput plant phenotyping.

Leaves are complex and highly sophisticated 3D geometries that have been optimized over the course of evolutionary time to balance water distribution, photosynthesis, and structural integrity, among many other biological functions. However, imaging technology has only recently enabled a clear view and, more importantly, the capacity to digitally represent leaf 3D anatomy (Théroux‐Rancourt et al., 2017). Today, 3D imaging permits precise spatial measurement and biophysical modeling of leaf internal geometry that can deliver novel insights about basic leaf function, such as CO_2_ transport (Ho et al., 2016; Lehmeier et al., 2017; Earles et al., 2018, 2019; Lundgren et al., 2019), H_2_O transport (Scoffoni et al., 2017), and mechanical structure (Pierantoni et al., 2019). Embracing the 3D complexity of leaf geometry permits us to understand when dimensionality reduction is tolerable and will ultimately guide more precise mechanistic scaling from tissue to crop and/or ecosystem.

Computationally, 3D imaging often produces large data sets (>20 GB) with hundreds to thousands of digital cross sections that do not immediately yield biologically relevant information. Regardless of the imaging modality, 3D images must be subsequently processed to extract biologically relevant information, such as tissue type, chemical composition, and material type. In the case of X‐ray microcomputed tomography (microCT) applied to plant leaves, this has led to the 3D description of the complex organization of the mesophyll cells and their surface area (Ho et al., 2016; Théroux‐Rancourt et al., 2017), and the description of novel anatomical traits related to the intercellular airspace (Lehmeier et al., 2017; Earles et al., 2018). Tissue segmentation can be done quickly using both proprietary and open source software via 3D thresholding based on pixel intensity values. However, in the case of leaf microCT scans, pixel intensity can primarily, and most often solely, distinguish between water‐filled cells and air‐filled void areas. As such, quick segmentations can generally only label cells and airspace, especially when using phase‐contrast reconstruction (Théroux‐Rancourt et al., 2017), resulting in the different tissues of a leaf (e.g., the epidermis, the mesophyll cells, the bundle sheaths, and the veins) being grouped together. Using this method, there is not a clear distinction between the background and the intercellular airspace. This results in segmentations being limited to small leaf volumes consisting solely of mesophyll cells and airspace to estimate leaf porosity and cell surface area, traits that are commonly measured when related to photosynthetic efficiency (e.g., Ho et al., 2016). However, small leaf volumes do not necessarily represent the whole leaf trait average, and thus a larger volume including veins is needed to limit sampling bias. To separate the leaf from the background and segment the different tissues within the leaf, current applications generally rely on the onerous process of hand segmentation, i.e., drawing with a mouse or a graphic tablet over single slices of a microCT scan to delimit and assign a unique value to each of the different tissues, either slice‐by‐slice or through the interpolation between different delimited regions throughout the scan (Théroux‐Rancourt et al., 2017; Harwood et al., 2020). As a result, studies incorporating 3D microCT data sets have been limited to smaller scanning endeavors, and the low replicability of these studies limits the impact of conclusions therein. Hand segmentation, as described above, can take up to one day of work for a coarse‐scale segmentation of tissues other than mesophyll cells and airspace (as in Théroux‐Rancourt et al., 2017). Furthermore, highlighting natural variations in size and curvature of the various tissues can substantially increase hand segmentation time (see, for example, Harwood et al. [2020] on a similar issue using serial block‐face scanning electron microscopy). Hence, segmentation is currently a major bottleneck in the use of this technology.

Machine learning presents an opportunity to substantially accelerate the image segmentation process for plant biological applications. Conventional computer vision techniques rely on a human to engineer and select visual features, such as shape, pixel intensity, and texture, that ultimately guide the underlying segmentation process. On the other hand, machine learning–based image processing allows the machine to directly select or engineer visual features (e.g., Çiçek et al., 2016; Berg et al., 2019). Machine learning–based image processing techniques fall along a continuum of unsupervised to supervised learning, which defines the degree to which the machine uses ground‐truth data for guiding its optimization function. Given the large number of images generated during an X‐ray microCT scan, machine learning–based image processing could lead to major efficiency gains in terms of human effort, enabling higher sample throughput and more complete data utilization as outlined above. In this study, we present a random forest machine learning framework for image segmentation of single X‐ray microCT plant leaf scans and test its performance on a grapevine leaf scan. We finally demonstrate how the rich 3D output can be used to extract biologically meaningful metrics from these segmented images.

## METHODS AND RESULTS

### Random forest segmentation and leaf traits analysis pipeline

**Figure 1:**
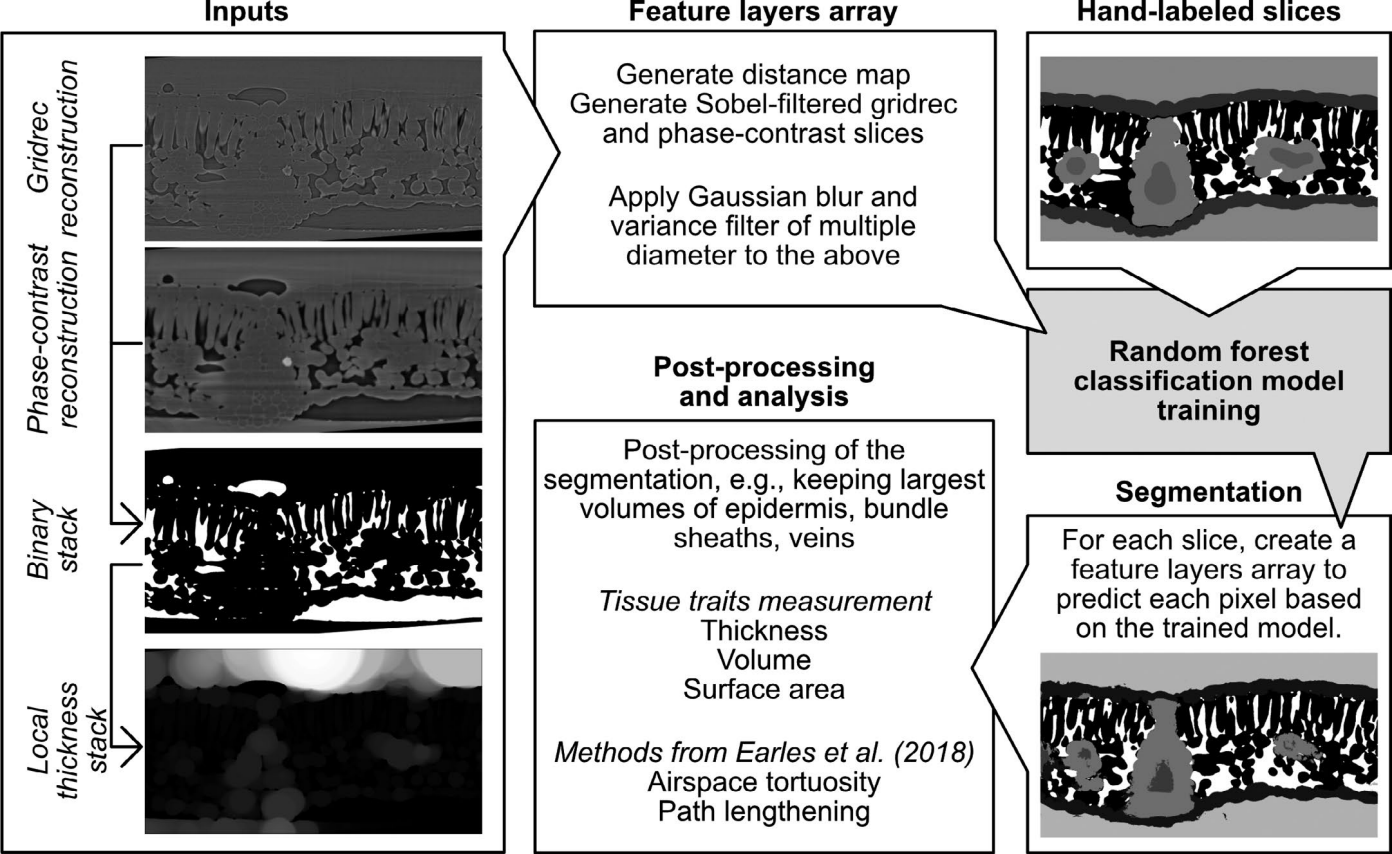
Schematic of the segmentation and analysis pipeline. Reconstructed microCT scans are manually thresholded to find the best value to segment the airspace of the leaf (as in Théroux‐Rancourt et al., 2017). Using this binary stack, a local thickness stack is created, which identifies for each pixel the diameter of the largest sphere contained in that area (lighter pixel values mean larger diameters). These input stacks are used to generate the feature layers arrays needed, along with the hand‐labeled slices, for the random forest classification model training. With the trained model, the complete stack of images is predicted, and from this predicted stack the image is post‐processed to remove false classifications, and leaf traits are analyzed. Note that all images are from the same position within the stack (i.e., same slice) except for the segmented image—using images from the same slice used for hand labeling provides identical segmentation.

The following pipeline was built for our projects using X‐ray synchrotron‐based microCT imaging and uses the freely available and open source software ImageJ (Schneider et al., 2012). We implemented this framework using the Python programming language for machine learning segmentation and for image analysis (an in‐depth user manual is available at https://github.com/plant‐microct‐tools/leaf‐traits‐microct; see Data Availability). Synchrotron‐based imaging allows reconstruction of the scans using the gridrec reconstruction, which reflects X‐ray absorption and provides a sharp but low‐contrast image highlighting the interface between cells (Dowd et al., 1999), and the phase‐contrast reconstruction, providing images with increased contrast between material of different absorptance (Paganin et al., 2002). Both reconstructions were at the base of our previous method (Théroux‐Rancourt et al., 2017). In its current state, the program requires the gridrec and phase‐contrast reconstructions in 8‐bit depth (Fig. 1). To prepare for model training and automated segmentation, one needs to prepare hand‐labeled slices. Briefly, using ImageJ, we first binarize (i.e., convert to black and white) the two reconstructions by applying a threshold, where grayscale values below are considered air and values above are considered cells (Fig. 1). Those two binary stacks are combined together as in Théroux‐Rancourt et al. (2017). Hand labeling is then done directly in ImageJ by drawing around each tissue, repeating the labeling over the desired number of slices while taking care to cover a range of anatomical variations such as the density and orientation of veins, which is the most important cause of variation between slices (Théroux‐Rancourt et al., 2017). In the current case, the background, both epidermises, and the bundle sheaths and vein pairs (1–3 per slice, each tissue labeled separately) were labeled by a single individual, and consistency was validated by G.T.R. (Fig. 1; see also Appendix S1 for the number of pixels per class). For a detailed methodology on preparing hand‐labeled slices, please refer to the public repository of this program on GitHub (see Data Availability).

The random forest classification model can then be trained using the hand‐labeled slices. We built our own framework on top of Python 3 and existing open source libraries such as numpy (Oliphant, 2006), scikit‐image (van der Walt et al., 2014), and scikit‐learn (Pedregosa et al., 2011). The image processing and random forest classification is summarized in Fig. 1. First, using the gridrec and phase‐contrast reconstructions, the program creates a binary image (as previously defined) using the threshold values for both stacks as input variables. This binary image is then used to create a local thickness map, which identifies through distance transformation and for each pixel the diameter of the largest sphere contained in that area, i.e., representing an estimate of the axis length or diameter of that structure or cell. This map provides additional information needed to predict class based on the object’s 3D size. Feature layer arrays are then built by applying a Gaussian blur or a variance filter, both of different diameters, to the gridrec and phase‐contrast slices. The same filters are also applied to a map of the distance from the top and bottom edge of the image to its center, and to gridrec and phase‐contrast slices that have been Sobel filtered to emphasize edges. These feature layer arrays are then used, along with the local thickness map, to train the random forest classification model by predicting pixel values on the desired number of hand‐labeled training slices, which are randomly selected within the full hand‐labeled stack. The framework will always fit a new random forest, and by default will use 50 estimators, but this can be adjusted by the user. Further parameters used to train the random forest model include whether to use out‐of‐box samples to estimate accuracy and whether to reuse the solution of the previous call to fit and add more estimators to the ensemble.

After training the model and testing its accuracy on the hand‐labeled slices, it is able to automatically predict the remaining slices (generally >1500) of the microCT scan in just a few hours; this represents a massive improvement over conventional methods, which require hundreds of hours of manual work (Théroux‐Rancourt et al., 2017). In this article, we present results for one single scan. If users intend to segment multiple scans, the procedure above (i.e., hand labeling and model training) would have to be done for each scan, as the framework currently provides suitable and accurate segmentations for plant anatomy analysis when trained on each scan.

The full stack prediction can then be passed on to the leaf traits analysis pipeline. A first step is to identify all tissues and apply post‐prediction correction to remove some false predictions that would bias biological trait analysis. In the case of laminar leaves, this includes identifying the two largest epidermis structures of similar volumes, as the abaxial and adaxial epidermis should each form a single volume within the stack. Thus, smaller volumes labeled as epidermis (e.g., identified within the mesophyll cells) are not considered to be actual epidermis and are removed during this correction. For veins and bundle sheaths, as they are also usually highly connected to each other, small volumes do not represent actual tissues and therefore very small and unique volumes (i.e., generally below 27 px^3^) are removed. Hence, the stack used for trait analysis was corrected to include only biologically relevant volumes.

From this corrected stack, biological metrics were computed. Here, we measured the thickness at each point along the leaf surface for the whole leaf, the abaxial and adaxial epidermis, and the whole mesophyll (leaf without the epidermis); standard deviation was determined for all metrics. We also measured the volume of all segmented tissues using a voxel count, as well as the surface area of the mesophyll cells connected to the airspace using a marching cube algorithm. Further analysis of the airspace can be made to compute tortuosity and path lengthening using a Python version of Earles et al. (2018) methods, which are not included in the current methods analysis.

### Testing the segmentation program

To test the performance of the segmentation program, we used the microCT scan of a ‘Cabernet Sauvignon’ grapevine (*Vitis vinifera* L.) leaf acquired at the TOMCAT tomographic beamline of the Swiss Light Source at the Paul Scherrer Institute in Villigen, Switzerland. Samples were prepared for microCT scanning as in Théroux‐Rancourt et al. (2017), the sample was mounted between pieces of polyimide tape and fixed upright in a styrofoam holder, and 1801 projections of 100 ms were acquired at 21 keV over 180° total rotation using a 40× objective, for a final pixel size of 0.1625 µm. The scans were reconstructed with the gridrec and paganin algorithms using the reconstruction pipeline at the TOMCAT beamline. Twenty‐four slices spread evenly across the full 1920‐slice stack were hand labeled for epidermis, background, veins and bundle sheaths, mesophyll cells, and intercellular airspace as described above. To facilitate the testing, the *x* and *y* dimensions were halved, yielding a pixel size of 0.325 µm in those dimensions, but keeping the original dimensions of 0.1625 µm in the depth (*z*) dimension, hence reducing the file size by four, from approximately 6 GB down to 1.5 GB, a size easily handled by the program.

To understand the impact of training a model using different numbers of manually segmented slices, we iteratively trained the model using one through 12 slices. We repeated this process 30 times for each number of training slices using randomly selected training slices for each iteration. To cross‐validate between hand‐labeled ground truth and model predictions, each trained model was used to predict a test set consisting of all slices that were hand labeled but that had not been used to train the model (e.g., if six slices were used for training, the remaining 18 slices were predicted using the model). Confusion matrices were then created for each prediction test. Note that no post‐prediction corrections were applied and, as such, the results that follow present the raw predictions.

**Figure 2:**
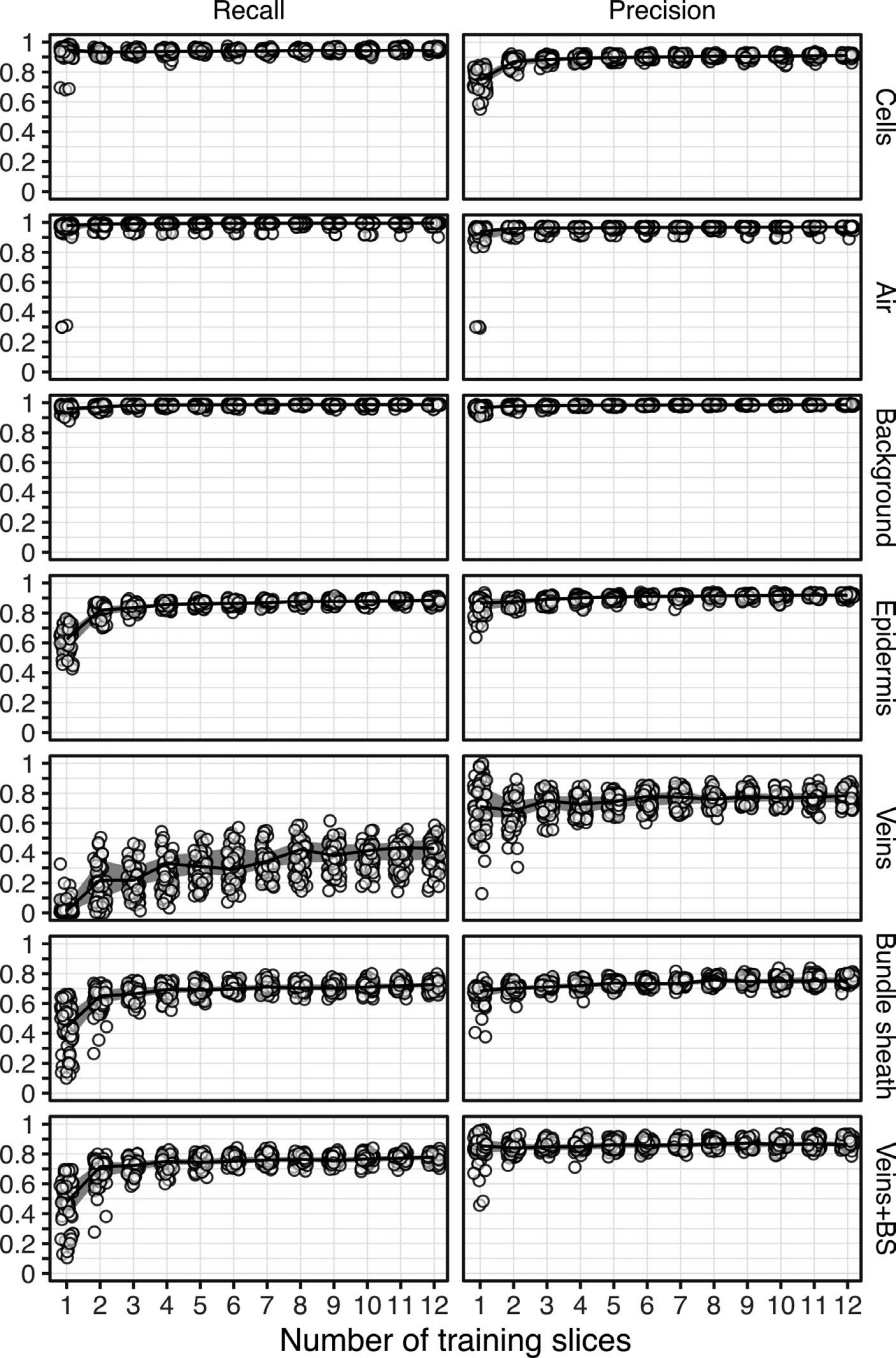
Recall (left) and precision (right) as a function of the number of training slices used to predict tissue classes in a microCT leaf scan (pixel size: 0.325 µm; 762,999 pixels per slice to predict). Circles represent raw values of individual predictions, solid lines represent the median value of 30 predictions per amount of training slices, and thick gray lines represent the region between the 25th and 75th quantiles.

From each confusion matrix, we evaluated precision and recall for each biological class (Fig. 2). In the context of automated information retrieval for microCT image segmentation, recall may be interpreted as the sensitivity of the trained model to a given pixel class (i.e., the portion of correctly identified pixels in a given class) relative to all pixels belonging to this class. On the other hand, precision represents the positive predictive value of the model within a given pixel class (i.e., the number of pixels correctly identified as belonging to a given class) divided by this value, plus the number of pixels falsely identified. It can be logically deduced why some people refer to recall as *quantity* of positive identification, and precision as the *quality* of positive identification.

In the mesophyll cell class, recall was generally >90% even when training on fewer than three manually segmented slices; this means that >90% of all mesophyll cell pixels were correctly identified as cells, suggesting the trained random forest model is highly sensitive to cells. The same can be said for the airspace and background classes, which plateaued at about 95% recall using more than one training slice. The trained models do not appear to be as sensitive to pixels of the epidermis class. Indeed, we observed a minimum of four training slices required to drive epidermis class recall above 90%. With vein and bundle sheath considered together as one class, at least four training slices were required to reach a maximum recall value of ~80%; the remaining 20% were false negatives (i.e., identified as other classes). Interestingly, when separating bundle sheath and vein into distinct classes, the bundle sheath class also reached a maximum recall value of ~75% using more than four training slices. Isolating the vein class from the bundle sheath class greatly impacted the trained model’s sensitivity to vein detection. Recall was not observed above 55%, and generally stayed under 40% unless the model was trained on more than eight manually segmented slices.

To achieve precision >90% in the airspace, background, and epidermis tissue classes, a minimum of two training slices should be used. Interestingly, training on more than two slices did not seem to translate to a substantial improvement in precision for these classes. However, observed precision for the mesophyll cell tissue class did not plateau until training on more than three slices. While the maximum precision for mesophyll cells was stably >90%, the lower precision values consistently observed when training on one or two slices (i.e., as low as 60%) suggest the software is not as reliable for this tissue class. It is therefore important to train on more than three slices if mesophyll cell traits are of interest. In the vein class, the software was observed to positively identify pixels at a rate of about 80% when trained on more than two slices. In other words, even though the software is not very sensitive to the vein class, it is quite reliable when it does make a positive identification in the vein class.

**Figure 3:**
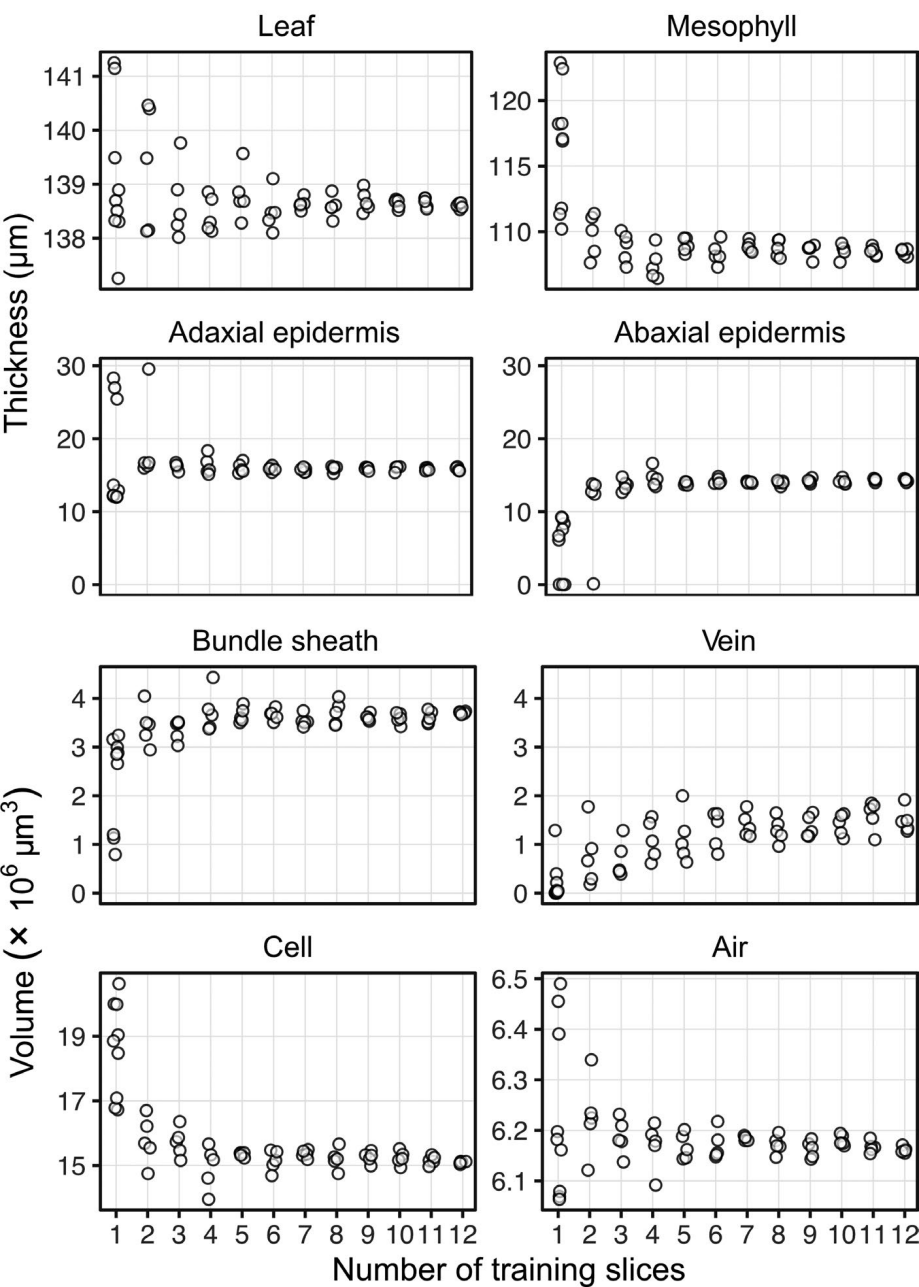
Variation in the measured tissue thicknesses and volumes based on the number of training slices used. False classification of inner leaf pixels as epidermis occurs more with one or two training slices, which resulted in the two epidermises being connected together, hence making the individual thickness estimates wrong. Standard deviations of the thickness estimates are presented in Appendix S2.

To evaluate how the number of training slices affected the measurement of biological traits, we used a subset of the 30 models per number of training slices to carry out predictions over the full stack instead of over the 24 hand‐labeled slices. We made five predictions over full stacks per number of training slices used, except for models trained with one slice, for which we carried out seven supplementary predictions to account for the expected variability in results. These full stack predictions were then passed through the leaf traits analysis program to extract relevant leaf anatomical traits (Fig. 3). Anatomical measures were the least constant between predictions when using one training slice. The most variable were the epidermis thickness estimates, with values ranging from near 0 µm for the abaxial epidermis to almost 30 µm in the adaxial epidermis, meaning that false segmentations of epidermis occurred between both epidermises such that they were connected and could not be automatically distinguished from one another as happened from three training slices onward. This false segmentation of the epidermis led to a highly variable whole mesophyll thickness (i.e., the leaf without the epidermis), which became less variable (<5%) when using at least three training slices. However, the overall leaf thickness was the least variable, with less than ~1.5‐µm variation (~1% total thickness) when using three or more training slices, a variation we consider equal or even lower than manual measures. This technique benefits greatly from measuring over each point, or voxel column, of the leaf area, allowing for the integration of millions of thickness measures, thus buffering local errors due to false segmentation. Volumetric anatomical traits became constant in variation and values at a minimum of five training slices for the bundle sheath, mesophyll cells, and airspace. As with precision and recall, vein volume substantially varied until about seven training slices, where values and variation plateaued. The volume of the leaf, the whole mesophyll, and epidermises each exhibited results similar to those for their thickness.

### How many slices should be hand labeled?

In the test presented above, the greater the number of total pixels represented by any class, the fewer training slices required to reach maximum sensitivity (i.e., recall). For example, the air, cells, and background classes are the most common pixel types and clearly show >90% sensitivity (recall) training on as few as two manually segmented slices (Fig. 2); with four manually segmented slices, different segmentations generated similar biological traits (Fig. 3). Veins and bundle sheaths are difficult to segment as they generally present very low contrast between each other and are difficult to distinguish both computationally and visually. In the usage we have made of the program (current study; Théroux‐Rancourt et al., 2020a), we are generally interested in defining where the vasculature (veins and bundle sheath together) is rather than in extracting traits related to those tissues, and as such six slices seem appropriate to obtain a reliable prediction of the volume of those two tissues (Figs. 2, 3). It is more problematic when a class is represented by a smaller number of pixels, as the number of training slices required to reach maximum sensitivity in this class increases (Fig. 2). This is an inherent class imbalance issue that cannot be solved because of the anatomy of the leaves; the number of training slices needed should allow to reach the desired precision and recall for the class or tissue with the lowest number of pixels per slice. For example, thinner tissues like the epidermis require a minimum of five training slices to reach constant precision, recall, and biological traits. As the imaged thickness or smaller axis of a tissue is dependent on the magnification used, care should be taken when planning a scanning endeavor to have sufficient magnification to acquire enough pixels per tissue or class of interest to facilitate subsequent segmentation. Using the testing procedure presented here on scans from previous scanning endeavors could help guide future microCT setups to acquire and extract high‐quality biological data.

## CONCLUSIONS

**Figure 4:**
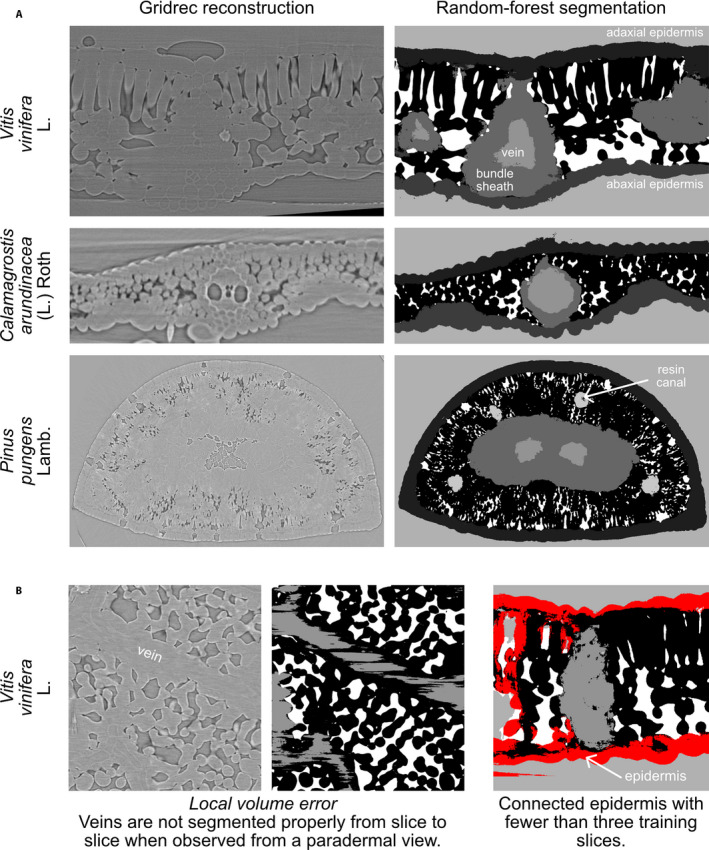
Random forest segmentation examples (A) for a grapevine leaf (*Vitis vinifera*), a grass (*Calamagrostis arundinacea*), and a pine needle (*Pinus pungens*), and common segmentation issues (B). Gridrec reconstructions on the same slices are shown on the left to compare with the predicted tissues based on random forest models trained on hand‐labeled slices. One of the main segmentation issues is the local volume problem, caused by 2D rather than 3D segmentation, which results in, for example, veins that are labeled on one slice and not on the other (shown by black areas in between gray‐labeled veins). Another issue is having the epidermis connected throughout the leaf at a small number of model training slices, here highlighted in red, where a volume might appear in 2D to be disconnected but is shown in 3D to be connected.

We present here an image segmentation framework using open source software that automatically segments image stacks of microCT scans consisting of thousands of single images and that requires only a few hand‐labeled single slices for each scan. This tool has allowed us to considerably speed up the segmentation of leaf scans while providing an increased level of detail: a well‐trained user can take less than one hour to prepare the model training slices needed to segment a whole 3D scan and extract relevant biological information. As a comparison, the coarse hand segmentation done in Théroux‐Rancourt et al. (2017) took about one full day of work for a pixel volume about a quarter of the size presented here. Furthermore, this segmentation and analysis pipeline has been successfully used on a variety of species and leaf forms (e.g., deciduous and evergreen laminar leaves, C_3_ grass leaves, conifer needles; Théroux‐Rancourt et al., 2020a), and is not limited to the tissues extracted above (e.g., resin canals in Fig. 4). Although it was not our objective to provide a universal tool to segment with a single model multiple leaf types, species, and scanning sessions, we consider this framework to have the ability and potential to be adapted and used on other plant material (e.g., different types of seeds, fruits, stems, and roots) to produce high‐quality segmentations.

However, this framework currently has limitations. For example, certain tissues are not evenly segmented, do not present the expected biological pattern, or present local volume errors, such as veins and bundle sheath constricting and expanding where they should be even from slice to slice. Using a slice‐by‐slice, 2D model training and segmentation approach can enhance this, and other machine learning methods may perform better on this front (e.g., Çiçek et al., 2016). However, we provide a simple tool that can be run on a regular workstation, without the requirement of special infrastructure such as a GPU cluster, for example. This tradeoff was acceptable for the majority of our work. Furthermore, models are currently generated for single scans and have yielded poor results when applied to other scans, even those of the same scanning sessions and the same species (i.e., similar settings and material). Again, this was an acceptable tradeoff as it significantly sped up the processing of microCT scans as mentioned above. Finally, other machine learning tools such as ilastik (Berg et al., 2019) and Trainable Weka Segmentation (Arganda‐Carreras et al., 2017) might require more upfront annotations to reach similar or better segmentations than the current pipeline; however, these tools may learn more broadly and therefore be able to apply one model to multiple scans and thus better exploit the potential of machine learning. The current pipeline is an acceptable compromise that can be used for specific tasks to provide accurate predictions with minimal annotation. However, when we used sparse annotations, ilastik and Trainable Weka Segmentation gave poor results on our images, most probably because of the limited or absence of gray value contrast between biologically relevant tissues, which further motivated the use of the current pipeline. Future milestones would be to implement 3D learning to better account for continuous and regular tissues, to make the trained model usable for similar scans (e.g., same sessions, species, and material), and to test the performance of other classifiers such as support vector machine, *k*‐nearest neighbors, and naive Bayes.

To conclude, this segmentation framework allowed us to generate a considerable amount of segmented leaves over a wide array of species (see Théroux‐Rancourt et al., 2020a). It has the potential to empower researchers to broaden sampling, to ask new questions about the 3D structure of leaves, and to derive new and meaningful metrics for biological structures.

## AUTHOR CONTRIBUTIONS

J.M.E. and M.R.J. conceptualized and programmed the random forest segmentation program, with assistance from G.T.R. G.T.R. programmed the leaf traits analysis and the segmentation testing, with assistance from M.R.J. G.T.R. acquired and processed the microCT test scan at the Swiss Light Source. E.J.F. tested and commented on the segmentation program during the development. G.T.R., M.R.J., and J.M.E. wrote the paper, with revisions from all authors. A.M. and C.R.B. provided funding and access to data.

## Supporting information


**APPENDIX S1.** Average proportion of pixels per tissue in the 24 slices of the training data set.

**APPENDIX S2.** Standard deviation of thickness estimates presented in Fig. 2.

## Data Availability

The code and an in‐depth user manual are available at https://github.com/plant‐microct‐tools/leaf‐traits‐microct. Future updates will be integrated to this repository. The microCT data set, training hand‐labeled slices, and all image outputs of the program including one full stack segmentation are available on Zenodo at https://doi.org/10.5281/zenodo.3694973 (Théroux‐Rancourt et al., 2020b).

## References

[aps311380-bib-0001] Arganda‐Carreras, I. , V. Kaynig , C. Rueden , K. W. Eliceiri , J. Schindelin , A. Cardona , and H. S. Seung . 2017 Trainable Weka Segmentation: A machine learning tool for microscopy pixel classification. Bioinformatics 33: 2424–2426. 28369169 10.1093/bioinformatics/btx180

[aps311380-bib-0002] Berg, S. , D. Kutra , T. Kroeger , C. N. Straehle , B. X. Kausler , C. Haubold , M. Schiegg , et al. 2019 ilastik: Interactive machine learning for (bio)image analysis. Nature Methods 16: 1226–1232. 31570887 10.1038/s41592-019-0582-9

[aps311380-bib-0003] Çiçek, Ö. , A. Abdulkadir , S. S. Lienkamp , T. Brox , and O. Ronneberger . 2016 3D U‐Net: Learning dense volumetric segmentation from sparse annotation *In* Ourselin S., Joskowicz L., Sabuncu M., Unal G., and Wells W. [eds.], Medical Image Computing and Computer‐Assisted Intervention – MICCAI 2016, 424–432. Lecture Notes in Computer Science, vol 9901. Springer, Cham, Switzerland.

[aps311380-bib-0004] Dowd, B. A. , G. H. Campbell , R. B. Marr , V. V. Nagarkar , S. V. Tipnis , L. Axe , and D. P. Siddons . 1999 Developments in synchrotron X‐ray computed microtomography at the National Synchrotron Light Source *In* Bonse U. [ed.], Proceedings of SPIE, Developments in X‐Ray Tomography II, 224–236. Society of Photographic Instrumentation Engineers, Bellingham, Washington, USA.

[aps311380-bib-0005] Earles, J. M. , G. Théroux‐Rancourt , A. B. Roddy , M. E. Gilbert , A. J. McElrone , and C. R. Brodersen . 2018 Beyond porosity: 3D leaf intercellular airspace traits that impact mesophyll conductance. Plant Physiology 178: 148–162. 30042212 10.1104/pp.18.00550 PMC6130031

[aps311380-bib-0006] Earles, J. M. , T. N. Buckley , C. R. Brodersen , F. A. Busch , F. J. Cano , B. Choat , J. R. Evans , et al. 2019 Embracing 3D complexity in leaf carbon–water exchange. Trends in Plant Science 24: 15–24. 30309727 10.1016/j.tplants.2018.09.005

[aps311380-bib-0007] Harwood, R. , E. Goodman , M. Gudmundsdottir , M. Huynh , Q. Musulin , M. Song , and M. M. Barbour . 2020 Cell and chloroplast anatomical features are poorly estimated from 2D cross‐sections. New Phytologist 225: 2567–2578. 31553810 10.1111/nph.16219

[aps311380-bib-0008] Ho, Q. T. , H. N. C. Berghuijs , R. Watté , P. Verboven , E. Herremans , X. Yin , M. A. Retta , et al. 2016 Three‐dimensional microscale modelling of CO_2_ transport and light propagation in tomato leaves enlightens photosynthesis. Plant, Cell & Environment 39: 50–61. 10.1111/pce.12590 26082079

[aps311380-bib-0009] Lehmeier, C. , R. Pajor , M. R. Lundgren , A. Mathers , J. Sloan , M. Bauch , A. Mitchell , et al. 2017 Cell density and airspace patterning in the leaf can be manipulated to increase leaf photosynthetic capacity. The Plant Journal 64: 1061. 10.1111/tpj.13727 PMC5725688 28963748

[aps311380-bib-0010] Lundgren, M. R. , A. Mathers , A. L. Baillie , J. Dunn , M. J. Wilson , L. Hunt , R. Pajor , et al. 2019 Mesophyll porosity is modulated by the presence of functional stomata. Nature Communications 10: 2825. 10.1038/s41467-019-10826-5 PMC6597550 31249299

[aps311380-bib-0011] Oliphant, T. E. 2006 A guide to NumPy. Trelgol Publishing, USA.

[aps311380-bib-0012] Paganin, D. , S. C. Mayo , T. E. Gureyev , P. R. Miller , and S. W. Wilkins . 2002 Simultaneous phase and amplitude extraction from a single defocused image of a homogeneous object. Journal of Microscopy 206: 33–40. 12000561 10.1046/j.1365-2818.2002.01010.x

[aps311380-bib-0013] Pedregosa, F. , G. Varoquaux , A. Gramfort , V. Michel , B. Thirion , O. Grisel , M. Blondel , et al. 2011 Scikit‐learn: Machine learning in Python. Journal of Machine Learning Research 12: 2825–2830.

[aps311380-bib-0014] Pierantoni, M. , V. Brumfeld , L. Addadi , and S. Weiner . 2019 A 3D study of the relationship between leaf vein structure and mechanical function. Acta Biomaterialia 88: 111–119. 30779998 10.1016/j.actbio.2019.02.023

[aps311380-bib-0015] Schneider, C. A. , W. S. Rasband , and K. W. Eliceiri . 2012 NIH Image to ImageJ: 25 years of image analysis. Nature Methods 9: 671–675. 22930834 10.1038/nmeth.2089 PMC5554542

[aps311380-bib-0016] Scoffoni, C. , C. Albuquerque , C. R. Brodersen , S. V. Townes , G. P. John , M. K. Bartlett , T. N. Buckley , et al. 2017 Outside‐xylem vulnerability, not xylem embolism, controls leaf hydraulic decline during dehydration. Plant Physiology 173: 1197–1210. 28049739 10.1104/pp.16.01643 PMC5291720

[aps311380-bib-0017] Théroux‐Rancourt, G. , J. M. Earles , M. E. Gilbert , M. A. Zwieniecki , C. K. Boyce , A. J. McElrone , and C. R. Brodersen . 2017 The bias of a two‐dimensional view: Comparing two‐dimensional and three‐dimensional mesophyll surface area estimates using noninvasive imaging. New Phytologist 215: 1609–1622. 28691233 10.1111/nph.14687

[aps311380-bib-0018] Théroux‐Rancourt, G. , A. B. Roddy , J. M. Earles , M. E. Gilbert , M. A. Zwieniecki , C. K. Boyce , D. Tholen , et al. 2020a Maximum CO_2_ diffusion inside leaves is limited by the scaling of cell size and genome size. bioRxiv 2020.01.16.904458 [Preprint]. Published 16 January 2020 [accessed 2 July 2020]. Available from: 10.1101/2020.01.16.904458. PMC7934972 33622134

[aps311380-bib-0019] Théroux‐Rancourt, G. , M. R. Jenkins , J. M. Earles , C. R. Brodersen , A. J. McElrone , and E. J. Forrestel . 2020b Image dataset for “Digitally deconstructing leaves in 3D using X‐ray microcomputed tomography and machine learning.” Available at Zenodo repository. 10.5281/zenodo.3694973 [published 3 March 2020; accessed 13 July 2020]. PMC7394714 32765979

[aps311380-bib-0020] van der Walt, S. , J. L. Schönberger , J. Nunez‐Iglesias , F. Boulogne , J. D. Warner , N. Yager , E. Gouillart , and T. Yu . 2014 scikit‐image: Image processing in Python. PeerJ 2: e453. 25024921 10.7717/peerj.453 PMC4081273

