# Supplementary material for: Digitally deconstructing leaves in 3D using X‐ray microcomputed tomography and machine learning
Source: Appl Plant Sci. 2020 Jul 31;8(7):e11380. doi: 10.1002/aps3.11380 (PMC7394714; doi:10.1002/aps3.11380)
Supplement: Supplementary file 1 — APPENDIX S1. Average proportion of pixels per tissue in the 24 slices of the training data set. [file APS3-8-e11380-s001.docx]

**APPENDIX S1.** Average proportion of pixels per tissue in the 24 slices of the training data set.

| **Tissue type** | **No. of items^a^** | **Proportion of pixels in slice** | |
| --- | --- | --- | --- |
|  |  | **Average** | **Min–Max** |
| Background | 2 | 0.295 | 0.201–0.325 |
| Airspace | — | 0.131 | 0.089–0.182 |
| Mesophyll cells | — | 0.321 | 0.270–0.417 |
| Epidermis | 2 | 0.159 | 0.144–0.180 |
| Bundle sheaths | 1–4 | 0.082 | 0.051–0.132 |
| Veins | 1–4 | 0.014 | 0.005–0.042 |

^a^Number of separate items sharing the same label present in the slice. For airspace and mesophyll cells, generally all the pixels are connected, with many small regions disconnected.
