# Supplementary figures and images for: Digitally deconstructing leaves in 3D using X‐ray microcomputed tomography and machine learning
Source: Appl Plant Sci. 2020 Jul 31;8(7):e11380. doi: 10.1002/aps3.11380 (PMC7394714; doi:10.1002/aps3.11380)

**APPENDIX S2.** Standard deviation of thickness estimates presented in Fig. 2.

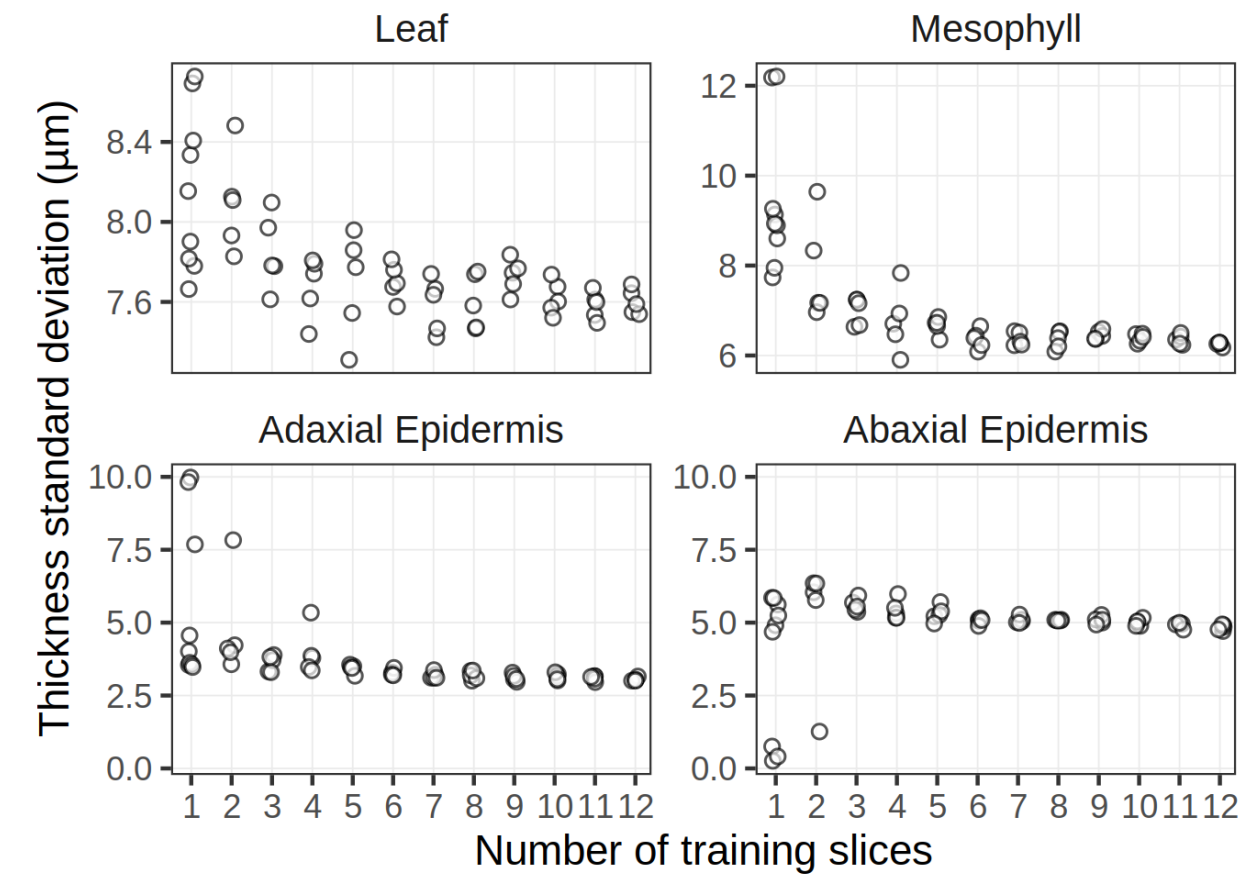

Supplement: Supplementary file 2 — APPENDIX S2. Standard deviation of thickness estimates presented in Fig. 2. [file APS3-8-e11380-s002.pdf]
